# Analysis of Risk Alleles and Complement Activation Levels in Familial and Non-Familial Age-Related Macular Degeneration

**DOI:** 10.1371/journal.pone.0144367

**Published:** 2016-06-03

**Authors:** Nicole T. M. Saksens, Yara T. E. Lechanteur, Sanne K. Verbakel, Joannes M. M. Groenewoud, Mohamed R. Daha, Tina Schick, Sascha Fauser, Camiel J. F. Boon, Carel B. Hoyng, Anneke I. den Hollander

**Affiliations:** 1 Department of Ophthalmology, Radboud University Medical Center, Nijmegen, the Netherlands; 2 Department of Epidemiology, Biostatistics and Health Technology Assessment, Radboud University Medical Center, Nijmegen, the Netherlands; 3 Department of Nephrology, Leiden University Nijmegen Medical Center, Leiden, the Netherlands; 4 Department of Ophthalmology, University Hospital of Cologne, Cologne, Germany; 5 Department of Ophthalmology, Leiden University Medical Center, Albinusdreef 2, Leiden, the Netherlands; 6 Department of Human Genetics, Radboud University Medical Center, Nijmegen, the Netherlands; Tufts University, UNITED STATES

## Abstract

**Aims:**

Age-related macular degeneration (AMD) is a multifactorial disease, in which complement-mediated inflammation plays a pivotal role. A positive family history is an important risk factor for developing AMD. Certain lifestyle factors are shown to be significantly associated with AMD in non-familial cases, but not in familial cases. This study aimed to investigate whether the contribution of common genetic variants and complement activation levels differs between familial and sporadic cases with AMD.

**Methods and Results:**

1216 AMD patients (281 familial and 935 sporadic) and 1043 controls (143 unaffected members with a family history of AMD and 900 unrelated controls without a family history of AMD) were included in this study. Ophthalmic examinations were performed, and lifestyle and family history were documented with a questionnaire. Nine single nucleotide polymorphisms (SNPs) known to be associated with AMD were genotyped, and serum concentrations of complement components C3 and C3d were measured. Associations were assessed in familial and sporadic individuals. The association with risk alleles of the age-related maculopathy susceptibility 2 (*ARMS2*) gene was significantly stronger in sporadic AMD patients compared to familial cases (p = 0.017 for all AMD stages and p = 0.003 for advanced AMD, respectively). *ARMS2* risk alleles had the largest effect in sporadic cases but were not significantly associated with AMD in densely affected families. The C3d/C3 ratio was a significant risk factor for AMD in sporadic cases and may also be associated with familial cases. In patients with a densely affected family this effect was particularly strong with ORs of 5.37 and 4.99 for all AMD and advanced AMD respectively.

**Conclusion:**

This study suggests that in familial AMD patients, the common genetic risk variant in *ARMS2* is less important compared to sporadic AMD. In contrast, factors leading to increased complement activation appear to play a larger role in patients with a positive family history compared to sporadic patients. A better understanding of the different contributions of risk factors in familial compared to non-familial AMD will aid the development of reliable prediction models for AMD, and may provide individuals with more accurate information regarding their individual risk for AMD. This information is especially important for individuals who have a positive family history for AMD.

## Introduction

Age-related macular degeneration (AMD) is a multifactorial disease and the leading cause of blindness among the elderly in developed countries.[1] With an ageing population, AMD is considered a major and growing health problem.[2] The disease, in its early stages, is characterized by drusen deposits and pigmentary abnormalities. Vision loss mainly occurs when the disease progresses to late AMD, which can be subdivided into geographic atrophy (GA) and choroidal neovascularization (CNV).[3]

Both environmental and genetic risk factors have been associated with the development and progression of AMD. The most consistently reported demographic and environmental risk factors are advanced age, high body mass index (BMI) and current cigarette smoking.[4–8]

Population-based analysis and twin studies have shown a strong genetic contribution to the development of AMD.[2,9–12] Major associations were reported for genetic variants in the complement factor H (*CFH*) and age-related maculopathy susceptibility 2 (*ARMS2*) genes.[3,13–17] Several pathways have been described to be implicated in the development of AMD, including the alternative complement pathway.[18,19] Genetic variants in several complement genes have been associated with AMD, including the *CFH*,[13–15,20] complement factor 3 (*C3*),[21–25] complement factor B (*CFB*),[24,26,27] and complement factor I (*CFI*) genes.[28]. Besides genetic variants in the complement genes, also systemic levels of complement components have been associated with AMD.[24,29,30]

Approximately 20% of AMD patients have a positive family history,[9–11,31] and first-degree relatives of AMD patients have an increased risk of developing AMD.[9,10,32] It has been suggested that the familial component of AMD may be explained by shared genetic or environmental factors.[10] However, the contribution of such factors in familial compared to non-familial AMD patients has not been studied comprehensively. We recently demonstrated that certain lifestyle factors, such as physical activity and red meat consumption, are significantly associated with AMD in sporadic cases but not in familial cases.[33] A recent study showed that the mean genotypic load of common AMD risk alleles in AMD families did not deviate significantly from genotypic loads predicted by simulation models.[34] However, the mean genotypic load in densely affected families was significantly lower than expected, suggesting such families may carry rare, highly penetrant genetic variants.[34] The purpose of this study is to investigate whether the contribution of common genetic variants differs between familial and non-familial AMD cases by interaction analyses. This will support the development of reliable prediction models for AMD, and may provide more accurate information regarding the individual risk for AMD, in particular for individuals who have family members with AMD and for whom this question is most urgent.

## Methods

### Subjects

In this study, we evaluated 2259 subjects, including 1216 AMD patients and 1043 control individuals from the Netherlands and Germany. All participants were derived from the European Genetic Database (EUGENDA, www.eugenda.org), an international database for molecular and clinical analysis of AMD. Subjects 50 years of age or older were included when information about gender, BMI, smoking behavior, and family history was available. In case subjects were related, only the first derived AMD patient and control subject of the family were included. Clinical data of their relatives were available in 68 families and were only used to determine the degree of reliability of the self-reported questionnaire. This study was approved by the local ethics committee on Research Involving Human Subjects of the RadboudUMC “Commissie Mensgebonden Onderzoek Regio Arnhem-Nijmegen” and met the criteria of the Declaration of Helsinki.

Before enrollment in the EUGENDA database, all subjects provided written informed consent and completed a detailed questionnaire on their medical history, family history of AMD, BMI, and lifestyle factors, such as smoking behavior. The study cohort was split into familial and sporadic subjects, based on the self-reported family history. A positive family history was defined as at least two first-degree relatives (parents and/or siblings) with AMD or possible AMD in a family. Participants with a positive family history were labeled as familial and participants without a positive family history were labeled as sporadic. Based on diagnosis and family history, the participants in this retrospective study were divided into four groups: unaffected individuals with a family history of AMD (referred to as familial controls) (n = 143), familial AMD cases (n = 281), unaffected individuals without a family history of AMD (referred to as sporadic controls) (n = 900), and sporadic AMD cases (n = 935). Familial cases were subdivided in patients with a mild (n = 184) or dense (n = 97) positive family history, where the latter group meets one of next 3 criteria: (1) both parents have (possible) AMD, or (2) one affected parent and at least 25% of the siblings are affected, or (3) at least 50% of the siblings are affected. Subjects with a mild positive family history did not meet any of these criteria. The BMI was subdivided in three groups: <25, 25–30 and >30 and smoking behavior was categorized into never, past and current smoking.

Each participant underwent digital color fundus photography performed after pupillary dilatation with topical 1.0% tropicamide and 2.5% phenylephrine. Both patients and controls also received spectral-domain optical coherence tomography (SD-OCT). Color fundus photographs and OCT scans of both eyes of all individuals were evaluated by two independent certified reading center graders according to the standard protocol of the Cologne Image Reading Center and Laboratory (CIRCL).[35] The diagnosis of AMD was defined as described previously,[36] based on the grading of the worst affected eye. AMD was classified by the presence of pigmentary changes together with at least 10 small drusen (<63μm) or the presence of intermediate (63–124 μm) or large drusen (≥125 μm diameter) in the Early Treatment Diabetic Retinopathy Study (ETDRS) grid. The subgroup of advanced AMD was defined as either AMD with subfoveal GA and/or CNV in at least one eye. Controls were classified as no abnormalities or only small drusen or pigmentary abnormalities.

### Genotyping

Venous blood was obtained for genetic analysis and the measurement of the complement components C3 and C3d. Complement component C3 and the activation fragment C3d were measured in serum samples as described previously.[29] The C3d/C3 ratio was calculated as a measure of complement activation. Genomic DNA was extracted from peripheral blood samples using standard procedures. Genotyping of nine single nucleotide polymorphisms (SNPs) known to be associated with AMD, in the *ARMS2* (rs10490924), *CFH* (rs1061170, rs800292, and rs12144939), *C3* (rs2230199 and rs1047286), *CFB* (rs4151667 and rs641153), and *CFI* (rs10033900) genes was performed in at least 85% of the included subjects with KASP™ genotyping assays (LGC Genomics) according to the manufacturer’s instructions. Genotype frequencies in the control individuals were tested for Hardy-Weinberg equilibrium.

### Statistical analysis

Standard descriptive statistics were used to describe baseline and clinical characteristics. To study differences in age (at participation), gender, BMI, smoking status, risk allele frequencies for AMD-associated SNPs, and complement levels between AMD patients and controls, multivariable logistic regression analyses were performed adjusted for the covariates age, gender, BMI and smoking status.

Differences in association of AMD-associated SNPs and complement levels in familial compared to sporadic AMD were analyzed with a multivariable logistic regression analysis, with correction for the covariates age, gender, BMI and smoking status. Statistical analyses were also performed with subdivision into mildly and densely affected families for factors which were significantly associated with familial AMD, to study the effect of AMD-associated SNPs and complement levels on the density of AMD in affected families.

Due to the skewed nature of the data, log-transformed values of the C3d/C3 ratios were used for analysis. Histograms of the distribution of the C3d/C3 ratio before and after log-transformation are shown in S1 Fig.

Two-sided p-values of less than 0.05 were considered statistically significant. Because multiple SNPs were analyzed and many tests of significance were performed in our study, Bonferroni correction was performed for the risk and interaction analysis of genetic factors. Data were analyzed using SPSS Software version 20.0 (SPSS Inc., Chicago, IL).

## Results

Baseline demographic data are depicted in Table 1. Increased age was a significant risk factor for AMD, in sporadic (Odds ratio (OR) 1.10; 95% Confidence Interval (CI) 1.09–1.11; p < 0.001) and familial patients (OR 1.17; 95% CI 1.13–1.21; p < 0.001). Female gender was not significantly associated with AMD in sporadic nor in familial cases. In sporadic patients the risk for AMD increased with increasing BMI (OR 1.45; 95% CI 1.05–1.99; p = 0.023), while BMI was not associated with AMD in familial patients. Current smoking was a significant risk factor for developing AMD in sporadic patients (OR 2.12; 95% CI 1.44–3.12; p <0.001), but was not significantly associated with AMD in familial patients.

**Table 1:** Demographics in familial and sporadic individuals.

		Total	Familial				Sporadic			
			AMD	Controls	P-value^†^	OR (95% CI)^†^	AMD	Controls	P-value^†^	OR (95% CI)^†^
**N(%)**		2259	281 (12.4)	143 (6.3)			935 (41.4)	900 (39.8)		
Mean age (SD)*		73.7 (8.2)	75.5 (7.9)	66.7 (6.8)	**<0.001**	**1.17 (1.13–1.21)**	76.6 (8.5)	71.3 (6.7)	**<0.001**	**1.10 (1.09–1.11)**
**Gender**	Male (%)	931 (41.2)	99 (35.2)	56 (39.2)	Ref		379 (40.5)	397 (44.1)	Ref	
	Female (%)	1328 (58.8)	182 (64.8)	87 (60.8)	0.801	1.06 (0.67–1.67)	556 (59.5)	503 (55.9)	0.086	1.20 (0.97–1.48)
**BMI**	<25.0 (%)	1033 (45.7)	137 (48.8)	67 (46.9)	Ref		426 (45.6)	403 (44.8)	Ref	
	25.0–30.0 (%)	948 (42.0)	110 (39.1)	58 (40.6)	0.701	0.91 (0.57–1.46)	386 (41.3)	394 (43.8)	0.572	1.06 (0.86–1.32)
	>30.0 (%)	278 (12.3)	34 (12.1)	18 (12.6)	0.983	1.01 (0.51–1.99)	123 (13.2)	103 (11.4)	**0.023**	**1.45 (1.05–1.99)**
**Smoking**	Never (%)	993 (44.0)	105 (37.4)	62 (43.4)	Ref		427 (45.7)	399 (44.3)	Ref	
	Past (%)	1075 (47.6)	147 (52.3)	70 (49.0)	0.142	1.42 (0.89–2.26)	415 (44.4)	443 (49.2)	0.868	0.98 (0.79–1.22)
	Current (%)	191 (8.5)	29 (10.3)	11 (7.7)	0.103	1.97 (0.87–4.24)	93 (9.9)	58 (6.4)	**<0.001**	**2.12 (1.44–3.12)**

Abbreviations: AMD = age-related macular degeneration; Familial = positive family history for AMD (confirmed or possible AMD in at least one close relative (parent, sibling or child))

Sporadic = negative family history for AMD; OR = odds ratio; CI = confidence interval; N = number of patients; SD = standard deviation; Ref = reference group; BMI = body mass index.

* Age at participation.

^†^ Adjusted for age, gender, body mass index and smoking status.

P-values and ORs printed in bold indicate significant associations.

In a subset of 68 families, clinical examination data of the siblings and parents were available. The self-reported family history of the probands was correct in 93% of these families. Only in 1 out of 68 subjects (1.5%) who reported in the questionnaire to have close relatives with (possible) AMD, none of the examined family members seemed to be affected on ophthalmological examination and therefore he was incorrectly classified as familial. In addition, 4 out of 68 subjects (6%) were incorrectly classified as sporadic. 56 probands reported a positive family history. Of those, 30 reported a densely positive family history, which was correct in 29 probands (97%). Only in one proband who reported AMD in one parent and in 1 out of 4 sibs, the densely positive family history was incorrect since no siblings had AMD at ophthalmic examination. The number of affected family members was correct in 66%, and an underestimation or overestimation of the number affected family members was reported in 27% and 7%, respectively.

The allele frequencies of AMD-associated SNPs and the differences in association with AMD (all stages) between familial and sporadic subjects are shown in Table 2. The *ARMS2* risk allele was a significant risk factor for AMD in sporadic cases (OR 2.49; 95% CI 2.12–2.93; p < 0.001). In familial cases this effect was also observed, albeit with a weaker effect (OR 1.60; 95% CI 1.16–2.22; p = 0.005). This difference in association was significant (p = 0.017). The *CFH* Y402H allele was significantly associated with AMD in both sporadic and familial cases (OR 1.81; 95% CI 1.57–2.09; and OR 2.20; 95% CI 1.58–3.06, respectively (p < 0.001)), and contrary to the *ARMS2* SNP, this association did not significantly differ between familial and sporadic patients. Other genetic variants in the *CFH*, *C3*, *CFB* and *CFI* genes were not significantly associated with AMD in both sporadic and familial cases. The serum C3d/C3 ratio, as a measure of the systemic activity of the complement system, was a significant risk factor for AMD among sporadic patients (OR 1.84; 95% CI 1.40–2.4; p = <0.001)but did not reach significance among familial patients (OR 2.10; 95% CI 1.14–3.87; p = 0.017) after correction for multiple testing. The difference in serum C3d/C3 levels between familial and sporadic subjects was not significant (p = 0.669).

**Table 2:** Risk estimates and risk differences of allele frequencies of AMD-associated SNPs and serum complement activation levels for all AMD grades based on family history.

	Total (N = 2259)	Familial/sporadic	Familial				Sporadic			
		P-value^†^	AMD	Controls	P-value^†^	OR (95% CI)^†^	AMD	Controls	P-value^†^	OR (95% CI)^†^
SNP / risk allele	N (%)		(N = 281)	(N = 143)			(N = 935)	(N = 900)		
**ARMS2** / rs10490924 / T (%)	2259 (100)	**0.017**	46.6	33.2	**0.005**	**1.60 (1.16–2.22)**	39.4	21.0	**<0.001**	**2.49 (2.12–2.93)**
**CFH Y402H**/rs1061170 / C (%)	2259 (100)	0.288	60	40.9	**<0.001**	**2.20 (1.58–3.06)**	50.6	35.4	**<0.001**	**1.81 (1.57–2.09)**
**CFH** / rs800292 /A (%)	1936 (85.7)	0.478	16.9	19.0	0.385	0.82 (0.53–1.28)	18.8	25.5	**<0.001**	**0.70 (0.58–0.83)**
**CFH** / rs12144939 / T (%)	1947 (86.2)	0.896	9.2	16.4	0.052	0.62 (0.38–1.01)	13.9	20.3	**<0.001**	**0.60 (0.49–0.73)**
**C3** / rs2230199 / G (%)	2254 (99.8)	0.848	28.1	23.5	0.148	1.31 (0.91–1.88)	23.5	20.4	0.007	1.26 (1.06–1.49)
**C3** / rs1047286 / A (%)	1952 (86.4)	0.556	28.6	21.1	0.052	1.48 (1.00–2.21)	22.8	19.6	**0.005**	**1.30 (1.08–1.56)**
**CFB** / rs4151667 / A (%)	2241 (99.2)	0.574	3.1	3.2	0.781	0.88 (0.36–2.15)	3.5	4.9	0.027	0.67 (0.47–0.96)
**CFB** / rs641153 / A (%)	1944 (86.1)	0.728	5.3	8.2	0.210	0.64 (0.32–1.28)	6.4	8.2	0.044	0.74 (0.55–0.99)
**CFI** / rs10033900 / T (%)	2227 (98.6)	0.260	49.1	44.7	0.193	1.25 (0.90–1.73)	50.7	49.2	0.851	1.01 (0.88–1.16)
**C3d/C3 ratio**	1840 (81.5)	0.669	4.47 (3.48–6.10)*	4.04 (3.16–5.43)*	0.017	2.10 (1.14–3.87)	4.46 (3.39–5.72)*	3.95 (3.01–5.21)*	**<0.001**	**1.84 (1.40–2.43)**

Abbreviations: AMD = age-related macular degeneration; Familial = positive family history for AMD; Sporadic = negative family history for AMD; OR = odds ratio; CI = confidence interval; N = number of patients.

* Median (interquartile range).

^†^ Adjusted for age, gender, body mass index and smoking status.

Missing genotypes were <15%. P-values and ORs printed in bold indicate significant associations after correction for multiple testing.

The allele frequencies of AMD-associated SNPs and the differences in association with advanced AMD between familial and sporadic subjects are shown in Table 3. The findings for advanced AMD were similar as for all AMD stages, although the ORs of the common variants were stronger than for all AMD stages. Also, the difference in association of the *ARMS2* allele in subjects with a positive family history compared to those with a negative family history was even stronger for the development of advanced AMD (p = 0.003). No other SNPs differed in association between familial and sporadic subjects with advanced AMD and neither did the C3d/C3 ratio.

**Table 3:** Risk estimates and risk differences of allele frequencies of AMD-associated SNPs and serum complement activation levels for advanced AMD based on family history.

	Total (N = 2259)	Familial/sporadic	Familial				Sporadic			
		P-value^†^	AMD	Controls	P-value^†^	OR (95% CI)^†^	AMD	Controls	P-value^†^	OR (95% CI)^†^
SNP / risk allele	N (%)		(N = 201)	(N = 143)			(N = 571)	(N = 900)		
ARMS2 / rs10490924 / T (%)	1815 (100)	**0.003**	50.3	33.2	**0.001**	**1.92 (1.33–2.79)**	46.6	21.0	**<0.001**	**3.63 (2.98–4.42)**
**CFH Y402H**/rs1061170 / C (%)	1815 (100)	0.875	64.2	40.9	**<0.001**	**2.66 (1.79–3.95)**	58.3	35.4	**<0.001**	**2.75 (2.30–3.30)**
**CFH** / rs800292 /A (%)	1522 (83.9)	0.373	12.8	19.0	0.089	0.60 (0.34–1.08)	13.9	25.5	**<0.001**	**0.45 (0.35–0.59)**
**CFH** / rs12144939 / T (%)	1532 (84.4)	0.824	7.2	16.4	0.011	0.44 (0.23–0.83)	11.6	20.3	**<0.001**	**0.40 (0.30–0.54)**
**C3** / rs2230199 / G (%)	1810 (99.7)	0.928	28.3	23.5	0.185	1.32 (0.87–2.00)	23.8	20.4	0.012	1.30 (1.06–1.59)
**C3** / rs1047286 / A (%)	1537 (84.7)	0.882	27.7	21.1	0.261	1.32 (0.82–2.12)	23.1	19.6	0.008	1.37 (1.09–1.73)
**CFB** / rs4151667 / A (%)	1799 (99.1)	0.466	3.1	3.2	0.690	0.81 (0.29–2.26)	3.1	4.9	0.008	0.53 (0.34–0.85)
**CFB** / rs641153 / A (%)	1529 (84.2)	0.949	5.2	8.2	0.294	0.64 (0.27–1.48)	5.5	8.2	0.022	0.62 (0.41–0.93)
**CFI** / rs10033900 / T (%)	1791 (98.7)	0.578	48.0	44.7	0.463	1.15 (0.79–1.66)	51.6	49.2	0.784	1.02 (0.87–1.21)
**C3d/C3 ratio**	1478 (81.4)	0.532	4.29 (3.52–5.77)*	4.04 (3.16–5.43)*	0.017	2.23 (1.15–4.30)	4.37 (3.38–5.70)*	3.95 (3.01–5.21)*	**0.001**	**1.76 (1.26–2.46)**

Abbreviations: AMD = age-related macular degeneration; Familial = positive family history for AMD; Sporadic = negative family history for AMD; OR = odds ratio; CI = confidence interval; N = number of patients.

* Median (interquartile range).

^†^ Adjusted for age, gender, body mass index and smoking status.

Missing genotypes were <17%. P-values and ORs printed in bold indicate significant associations after correction for multiple testing.

**Fig 1:**
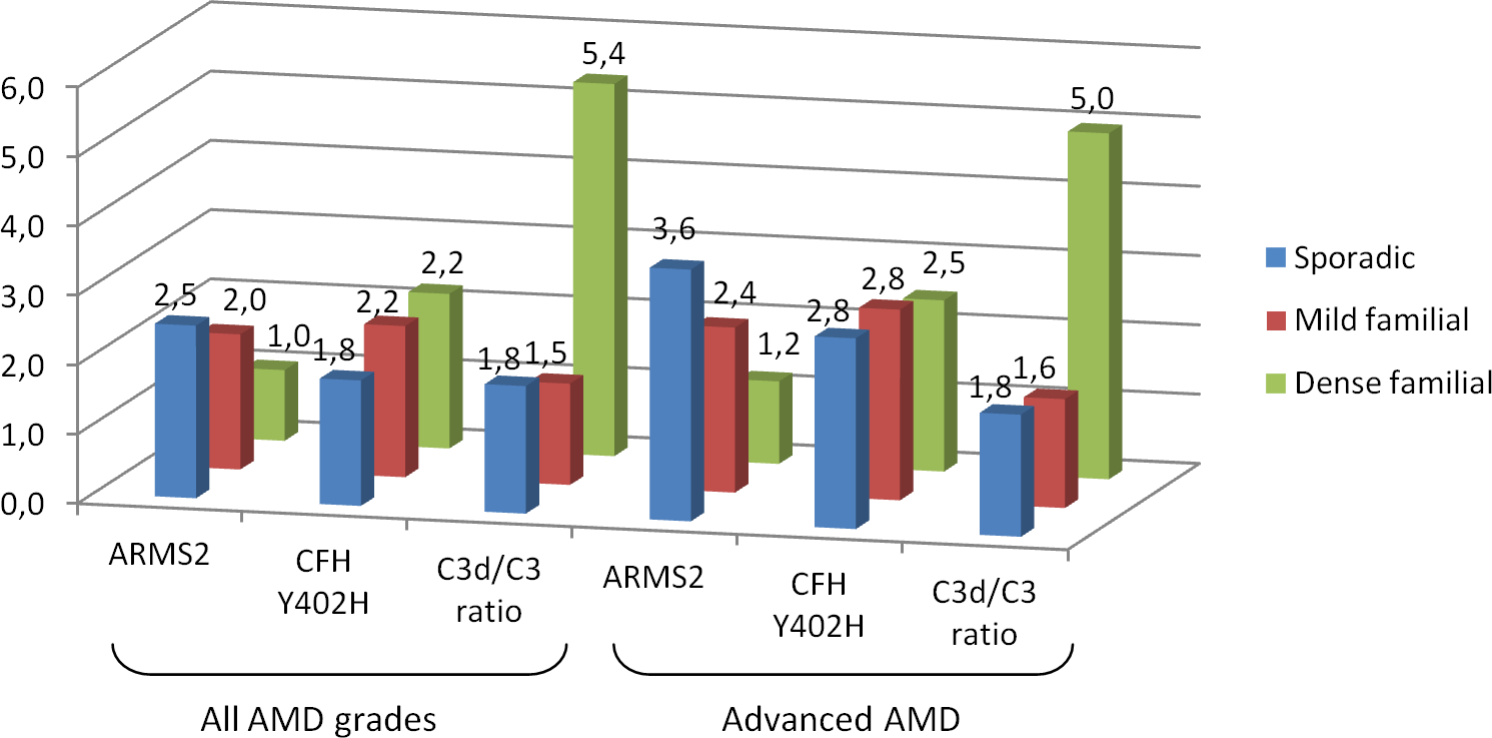
Odds ratios for risk variants in *ARMS2* and *CFH* and the C3d/C3 ratio for development of AMD split by family history. The risk variant in *ARMS2* confers a strong risk for AMD in the sporadic group. In the group with a dense family history there is no effect of this SNP. The *CFH* Y402H risk allele is associated with AMD in all subgroups, irrespective of family history. In case of a dense family history, the Log C3d/C3 ratio is associated with AMD development. In the subgroups with a mild family history, this effect was not observed. OR = odds ratio; AMD = age-related macular degeneration; Sporadic = negative family history for AMD; Familial = positive family history for AMD; Dense familial = a positive family history for AMD satisfying 1 out of 3 criteria: (1) both parents have (possible) AMD, or (2) one affected parent and at least 25% of number of the sibs are affected, or (3) at least 50% of the number of sibs is affected; Mild familial = a positive family history for AMD but in a lesser extent, not meeting one of the 3 criteria.

97 of the 281 familial AMD patients and 34 of the 143 familial controls reported a densely affected family history. The *ARMS2* SNP was not associated with AMD in patients from densely affected families, and this was significantly different from the association with sporadic AMD (p = 0.010 for all AMD stages and p = 0.002 for advanced AMD) (Table 4 and Fig 1). The association of the *CFH* Y402H allele with familial and sporadic AMD again did not differ. The C3d/C3 ratio showed the largest risk effect in patients with a densely affected family for all AMD (OR 5.37; 95% CI 1.54–18.69; p = 0.008) and advanced AMD (OR 4.99; 95% CI 1.41–17.68; p = 0.013) but this was not significantly different from the association with sporadic AMD.

**Table 4:** Risk estimates and risk differences of allele frequencies of *ARMS2* and *CFH* SNPs and serum complement activation levels in mild and densely affected AMD families.

	All AMD grades					Advanced AMD				
	Familial / sporadic	Familial		Sporadic		Familial / sporadic	Familial		Sporadic	
	P-value	P-value	OR (95% CI)	P-value	OR (95% CI)	P-value	P-value	OR (95% CI)	P-value	OR (95% CI)
**ARMS2** Mild familial	**0.010**	**0.001**	**1.95(1.31–2.92)**	**<0.001**	**2.49(2.12–2.93)**	**0.002**	**<0.001**	**2.38(1.49–3.80)**	**<0.001**	**3.63(2.98–4.43)**
Dense familial		0.946	1.02(0.58–1.81)				0.595	1.19(0.63–2.24)		
**CFH Y402H** Mild familial	0.575	**p<0.001**	**2.18(1.48–3.22)**	**<0.001**	**1.81(1.57–2.09)**	0.959	**<0.001**	**2.76(1.71–4.41)**	**<0.001**	**2.75(2.30–3.31)**
Dense familial		**0.014**	**2.23(1.18–4.23)**				**0.015**	**2.47(1.19–5.12)**		
**C3d/C3 ratio** Mild familial	0.199	0.296	1.46 (0.72–2.97)	**<0.001**	**1.84 (1.40–2.43)**	0.268	0.264	1.57 (0.71–3.45)	**0.001**	**1.76 (1.26–2.45)**
Dense familial		**0.008**	**5.37 (1.54–18.69)**				**0.013**	**4.99 (1.41–17.68)**		

AMD = age-related macular degeneration; Familial = positive family history for AMD; Sporadic = negative family history for AMD; OR = odds ratio; CI = confidence interval; Dense familial = a positive family history for AMD satisfying 1 out of 3 criteria: (1) both parents have (possible) AMD, or (2) one affected parent and at least 25% of number of the sibs are affected, or (3) at least 50% of the number of sibs is affected; Mild familial = a positive family history for AMD but in a lesser extent, not meeting one of the 3 criteria. All data are adjusted for age, gender, body mass index and smoking status; P-values and ORs printed in bold indicate significant associations.

## Discussion

In addition to environmental and genetic risk factors, a positive family history for AMD is an important risk factor for the development of AMD.[9,10,32] For a proper risk assessment it is therefore important to determine an individual’s family history for AMD. In this study we investigated whether the contribution of AMD-associated SNPs and C3d/C3 ratio differs between familial and non-familial AMD cases.

Our results show that the association of the *ARMS2* A69S genotype differed between familial and sporadic subjects. Within the group of cases and controls with a dense family history, *ARMS2* was not associated with AMD, whereas it was a strong risk factor for sporadic individuals. For the C3d/C3 ratio no significant difference was found between familial and sporadic subjects. However, in the subgroup with a dense family history, complement activation was most strongly associated with the presence of all AMD stages and advanced AMD.

The *ARMS2* A69S variant is one of the strongest genetic risk factors for AMD.[37] However, in densely affected families this risk variant seems to have less effect, and a high *ARMS2* risk allele frequency was found in controls with a positive family history. Testing the *ARMS2* SNP to estimate an individual’s AMD risk is thus more informative in patients without a positive family history. However, since both family history and SNPs are important factors in the development of AMD, and some discordance exists between risk estimates based on genetic testing and that based on family history analysis,[38] they should be used to complement one another in risk assessment. The fact that the family history for AMD affects the risk of the *ARMS2* genotype, suggests that there are other, unknown factors that increase the risk for AMD in the patients from densely affected families. This supports the theory that densely affected families may harbor rare, more penetrant genetic variants for AMD.[34,39,40] Even though no statistically significant difference was observed between familial and sporadic subjects concerning the association of the C3d/C3 ratio with AMD, the very high ORs that we reported for the patients from densely affected families can point towards a more important role for systemic complement activation in families with AMD compared to sporadic AMD patients. Risk alleles of *CFH* and *ARMS2* are independently associated with an increased C3d/C3 ratio,[29] and the higher complement level in familial AMD patients may (partly) be explained by the higher number of risk alleles of those SNPs in familial patients compared to sporadic patients. However, after additional adjustment for the *ARMS2* and complement SNPs, we determined that the estimated OR and corresponding CI for the C3d/C3 ratio did not significantly change. This further supports the hypothesis that rare, highly penetrant variants may contribute to the higher complement activation in familial AMD. Interestingly, several rare, highly penetrant AMD alleles have been described in several genes of the complement system,[39,41–44] and in densely affected families, mutations in the *CFH* gene have been identified.[40,43]

In this study no difference for the role of the *CFH* Y402H risk variant was observed between familial and sporadic subjects. Unlike *ARMS2*, the *CFH* Y402H risk SNP seems to be of equal importance for the development of AMD in both sporadic and familial individuals. This finding further underlines the important role of the complement system in familial AMD, both through common SNPs as well as rare genetic variants.

Four SNPs in the *ARMS2* and *CFH* genes were associated with AMD in sporadic cases in our study, but only the 2 major SNPs, *ARMS2* rs10490924 and *CFH* rs1061170, were also significantly associated with AMD in familial cases. The lack of association with the remaining 6 SNPs may due to the limited number of available subjects, and did not differ between familial and sporadic subjects. Stronger associations for advanced AMD compared to all AMD stages in sporadic cases indicate these risk SNPs play a more important role in the development of advanced stages of AMD than in the development of small and intermediate drusen. In sporadic AMD, an increased BMI and current smoking status showed a significant association with AMD in our study, which is in agreement with previous studies.[6–8,45] As these factors were not significantly associated with AMD in familial cases, environmental factors like smoking behavior and BMI may play a more important role in the development of AMD in sporadic patients than in familial cases. However, it should be noted that the absence of significant associations with AMD among familial subjects may be due to the limited number of available familial subjects.

The relatively low number of familial cases and controls is the main limitation of our study. This reduces the power of our analyses. However, after subdividing our familial dataset into mild and densely affected families we found that the differences in association between familial and sporadic cases were more pronounced, and this further underlines our findings. Nonetheless, our results should be interpreted with care and should be replicated in additional familial AMD cohorts in order to confirm our hypothesis.

In conclusion, this study demonstrates that the association of the *ARMS2* risk allele and complement activation levels in serum with AMD differs between familial and sporadic subjects. Our study suggests that *ARMS2* risk alleles have less effect in familial AMD patients than in sporadic AMD. In contrast, increased complement activation levels seem to play a larger role in patients with a dense positive family history compared to sporadic patients, which cannot be explained by known, common SNPs in the complement genes. A better understanding of factors that differ between individuals with and without a family history will aid the development of reliable prediction models for AMD, and may provide individuals with more accurate information regarding their individual risk for AMD. This information is especially important for individuals who have a dense positive family history for AMD.

## Supporting Information

S1 Fig Histograms showing distribution of C3d / C3 ratio before (A) and after (B) log-transformation.
(JPG)
