# Supplementary figures and images for: Analysis of Risk Alleles and Complement Activation Levels in Familial and Non-Familial Age-Related Macular Degeneration
Source: PLoS One. 2016 Jun 3;11(6):e0144367. doi: 10.1371/journal.pone.0144367 (PMC4892537; doi:10.1371/journal.pone.0144367)

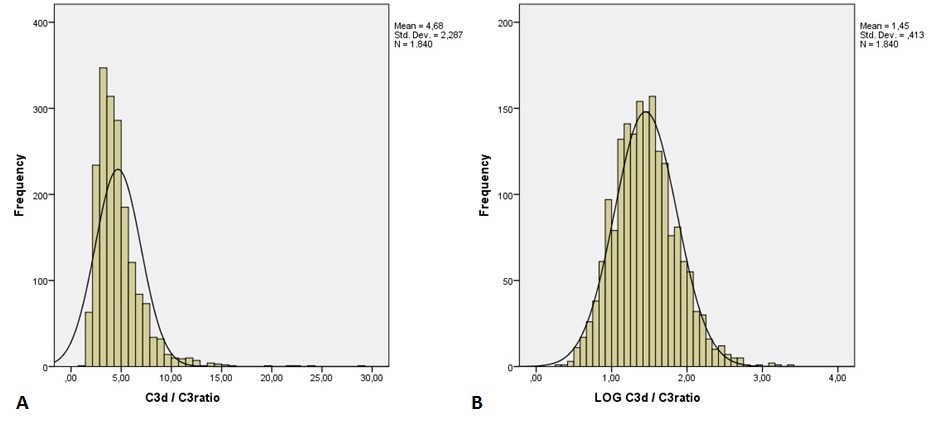

Supplement: S1 Fig — Histograms showing distribution of C3d / C3 ratio before (A) and after (B) log-transformation. (JPG) [file pone.0144367.s001.jpg]
